# Orbital apex syndrome due to invasive aspergillosis in an immunocompetent patient

**DOI:** 10.1016/j.idcr.2021.e01232

**Published:** 2021-07-24

**Authors:** Grace D. Cullen, Tara M. Davidson, Zachary A. Yetmar, Bobbi S. Pritt, Daniel C. DeSimone

**Affiliations:** a Department of Internal Medicine, Mayo Clinic, Rochester, MN, United States; b Division of Infectious Diseases, Mayo Clinic Rochester, MN, United States; c Division of Clinical Microbiology, Mayo Clinic, Rochester, MN, United States; d Department of Cardiovascular Diseases, Mayo Clinic, Rochester, MN, United States

**Keywords:** Orbital apex syndrome, aspergillus, Staphylococcus aureus, Immunocompetent

## Abstract

Infection is a rare cause of orbital apex syndrome (OAS) and most commonly occurs in immunocompromised hosts. We report a case of OAS in an elderly immunocompetent female due to invasive aspergillosis and *Staphylococcus aureus* co-infection. The patient required both surgical debridement and prolonged courses of antibiotic and antifungal therapy. Invasive fungal disease must be considered in cases of OAS, even in patients without classic risk factors.

## Case

**Fig. 1:**
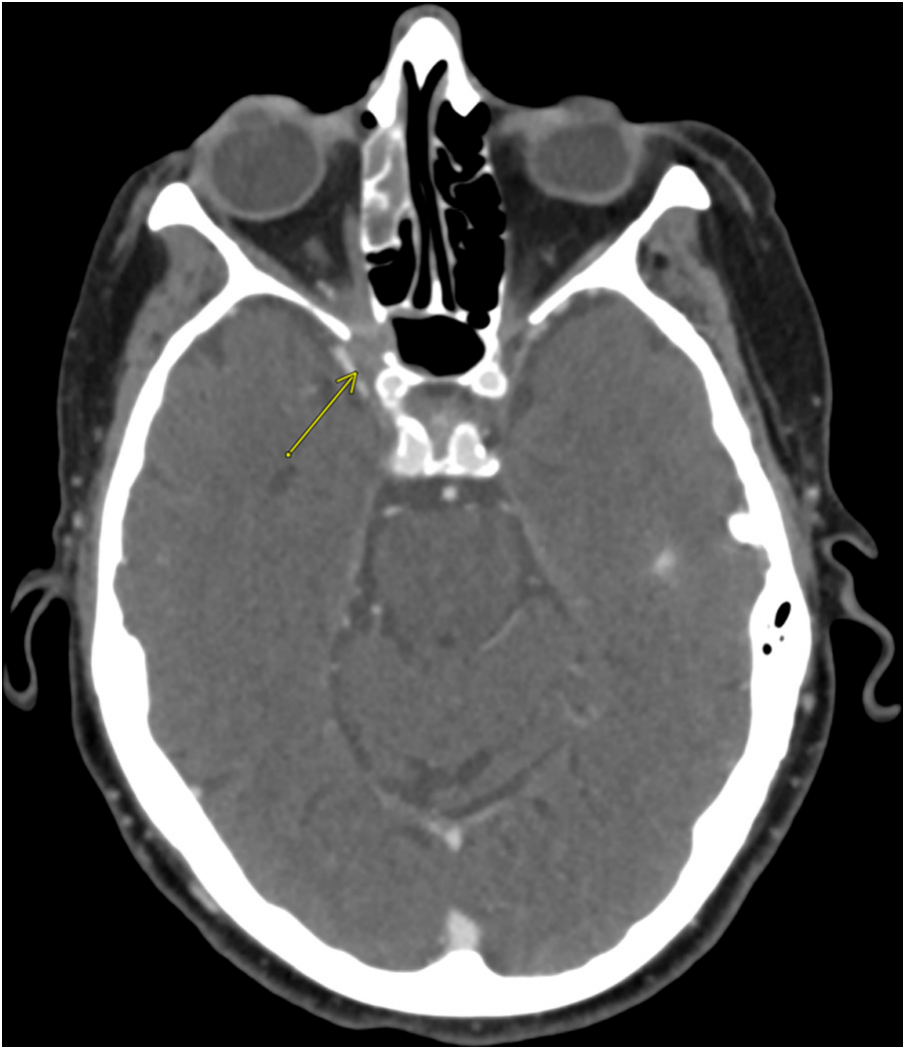
CT of the head and orbits with IV contrast demonstrating soft tissue thickening and indistinctness of fat in the region of the right optic canal and right orbital apex and an unusual bone defect in the right lateral wall of the right sphenoidal sinus.

**Fig. 2:**
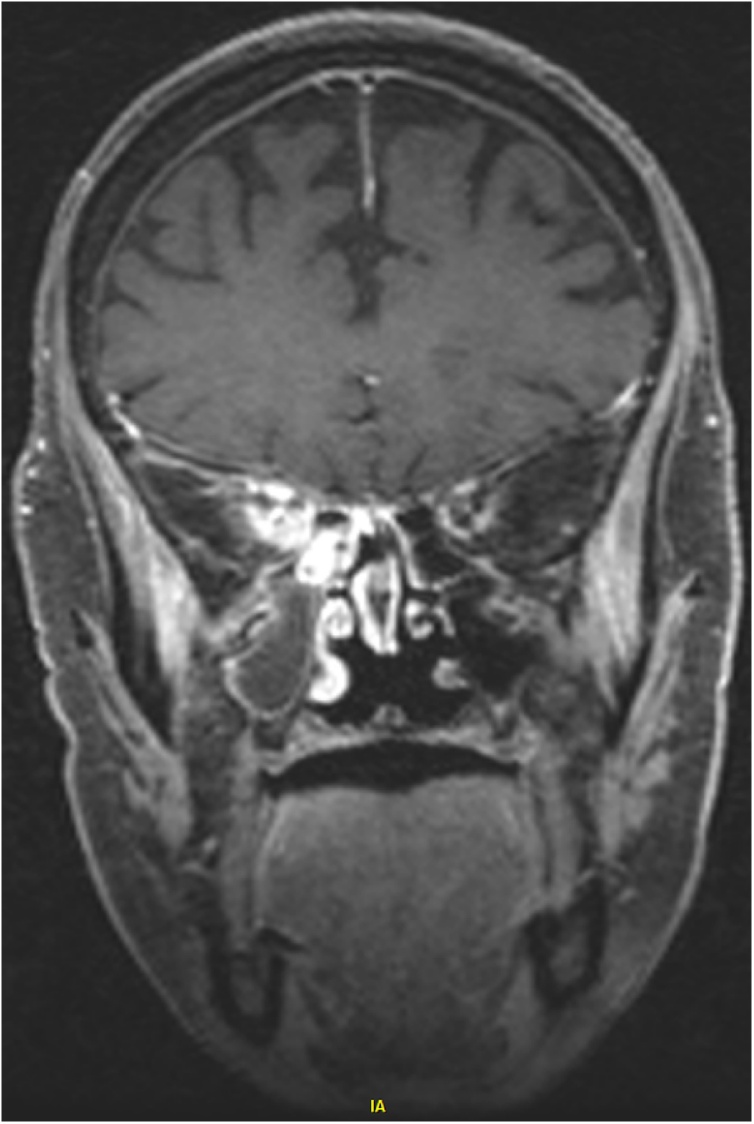
MRI of the brain, coronal view postcontrast T1 fat-suppressed CUBE, demonstrating phlegmonous extension of the right paranasal sinus infection into the orbital apex and toward the right cavernous sinus.

**Fig. 3:**
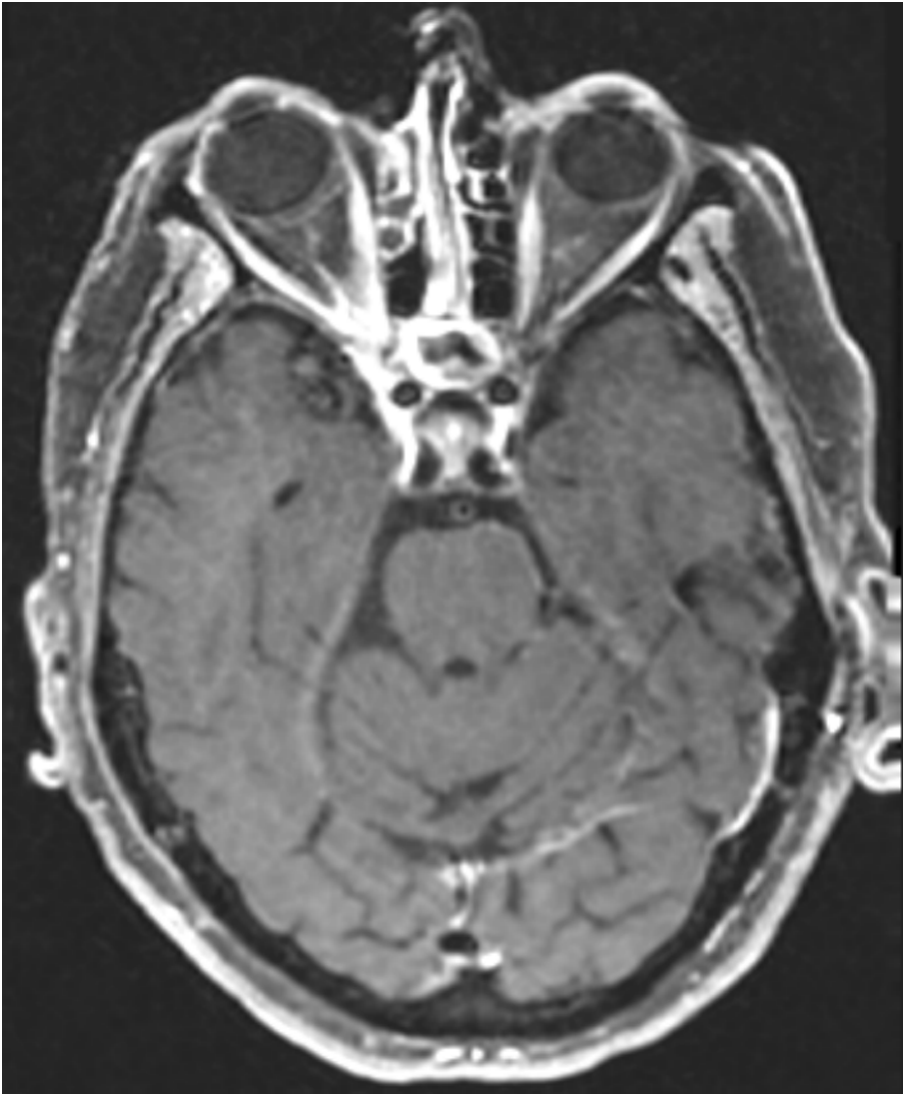
MRI of the brain, axial view postcontrast T1 fat-suppressed CUBE, demonstrating erosive defect in the right sphenoid sinus wall, allowing for phlegmonous extension of infection into right orbital apex.

An 86-year-old female with medical comorbidities including well-controlled type 2 diabetes (HbA1c 5.6 %) on oral therapy and glaucoma who presented with 2 weeks of right ear pain, right frontal headache and right facial pain just lateral to the nose. The patient also described painful extraocular movements (EOMs) on the right, decreased vision, and thick purulent drainage from the right nasal cavity. Vitals were normal with a blood pressure of 132/70, heart rate of 78, respiratory rate of 14, oxygen saturation of 97 % on room air, and temperature of 36.7 degrees Celsius. Examination was significant for hand motion vision, severely restricted right eye EOMs with ptosis, and excyclotorsion. Computerized tomography of the head and orbits demonstrated soft tissue thickening and indistinctness of fat in the region of the right optic canal and right orbital apex, possibly representing extension of paranasal inflammatory disease to an unusual bone defect in the right lateral wall of the right sphenoidal sinus (Fig. 1). Additionally, there was associated right anterior ethmoidal mucosal disease and complete opacification of the right maxillary antrum. Magnetic resonance imaging (MRI) of the brain without and with intravenous (IV) contrast showed right-sided paranasal sinus infection with transsphenoidal phlegmonous extension into the right orbital apex and cavernous sinus consistent with orbital apex syndrome (Fig. 2, Fig. 3). Ophthalmology and otolaryngology (ENT) were consulted and performed a right endoscopy of the nasal cavities. This revealed purulence from the right middle meatus obstructing the view of the sphenoid. Nasal swab was sent for bacterial culture. Infectious disease was consulted who recommended IV vancomycin, cefepime, and metronidazole in addition to dexamethasone.

**Fig. 4:**
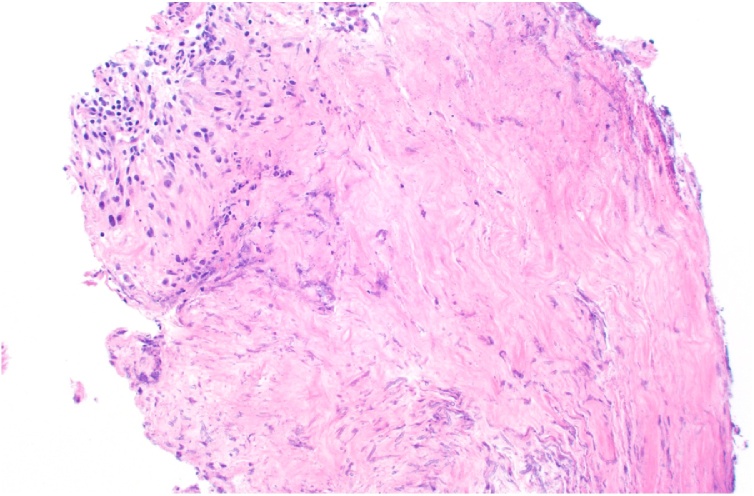
Right orbital apex tissue showing extensive necrosis and chronic inflammation (hematoxylin and eosin, 200x original magnification). A focus of faintly-staining, basophilic (purple-staining) invasive hyphae can be observed at the inferior portion of the tissue in this image (For interpretation of the references to colour in this figure legend, the reader is referred to the web version of this article).

**Fig. 5:**
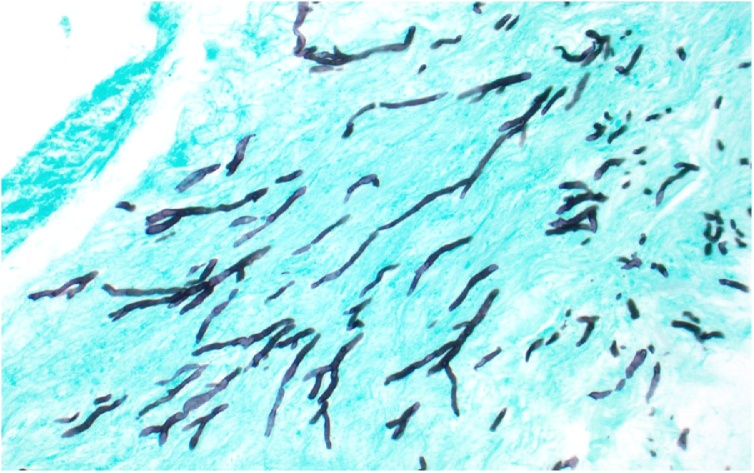
Gomori methenamine silver (GMS) staining for fungi of the tissue shown in Fig. 2 highlights the numerous, narrow, regularly septate hyphae with acute angle branching consistent with the cultured isolate of *Aspergillus fumigatus* (GMS, 400x original magnification).

The patient underwent surgery on hospital day three. ENT completed a broad endoscopic sinus debridement including right total ethmoidectomy, frontal sinusotomy, sphenoidotomy, tissue biopsy, and washout of orbital apex with tissues sent for bacterial and fungal cultures. Tissue from the right naris and right ethmoid sinus grew methicillin-susceptible *Staphylococcus aureus* (MSSA). Initial pathology at frozen section examination revealed mixed acute and chronic inflammation at all biopsy sites. The patient’s antibiotics were narrowed to IV cefazolin on hospital day 5. On hospital day 6, one colony of *Aspergillus fumigatus* grew from a culture of the right orbital apex tissue. The pathology revealed fungal hyphae consistent with *Aspergillus* spp. and related molds, with tissue invasion in the orbital apex (Fig. 4, Fig. 5). She was started on IV voriconazole. She was discharged to a skilled nursing facility after a 9-day hospital course with antimicrobials including oral cefdinir for a 4-week course and oral voriconazole with a planned treatment course of at least 12 weeks. At two-week follow-up with infectious disease, a repeat MRI brain showed worsening right ethmoid and sphenoid sinus fungal infection. Intravenous caspofungin was added to her regimen for a two-week course of therapy with clinical improvement. She underwent further debridement with rigid endoscopy at two, four, and six weeks after diagnosis with mostly crusted blood products and no evidence of necrotic tissue. Ophthalmologic exam ultimately demonstrated improvement in visual acuity to 20/50 with full EOMs.

## Discussion

OAS is a rare disorder characterized by a complex of cranial nerve deficits involving cranial nerves II, III, IV, V_1_, and VI associated with progressive vision loss, ophthalmoplegia, proptosis, ptosis, and periorbital pain [1,2]. Potential etiologies include infectious, malignant, autoimmune, and trauma, among others [1]. Infectious causes are most commonly due to orbital or post-septal cellulitis secondary to sinusitis, either by contiguous or perivascular spread [1]. The most common organisms are *Staphylococcus* spp., *Streptococcus* spp., and Gram-negative organisms such as *Pseudomonas aeruginosa* and *Klebsiella* spp., though fungal, viral, and parasitic infections have been documented [3]. OAS due to invasive fungal infection is uncommon and most often caused by *Aspergillus* or Mucorales spp., particularly in those with immunocompromising conditions or uncontrolled diabetes mellitus. However, cases in immunocompetent individuals have been reported [2,[4], [5], [6], [7], [8]]. Urgent treatment with both antimicrobials and surgery is necessary to avoid complications such as complete vision loss and cavernous sinus thrombosis [1]. Even with prompt treatment, infection can be fatal so a high index of suspicion is necessary [2].

Our patient is elderly, but an otherwise immunocompetent, non-neutropenic individual who presented with an indolent course of vision loss and periorbital pain and was subsequently diagnosed with OAS. Findings of invasive aspergillosis were unexpected, particularly given the alternative organism present and lack of traditional risk factors [9]. Her underlying diagnosis seems consistent with a chronic rather than acute invasive fungal sinusitis, which does tend to occur more in the immunocompetent population [10]. While she did have diabetes mellitus, this was well-controlled. Her advanced age and possible immunosenesence may have been predisposing factors for development of OAS [11].

## Conclusion

Patients presenting with features of OAS require a multidisciplinary team for optimal medical and surgical management. Prompt treatment with broad spectrum antimicrobials is crucial, and a high clinical suspicion for antifungal therapy is warranted, even in those without traditional risk factors for invasive fungal disease. Surgical debridement is necessary for source control and a prolonged course of antimicrobial therapy may be indicated if there is concern for residual disease.

## Author contribution

Grace Cullen – writing, editing.

Tara Davidson – writing, editing.

Zachary Yetmar – writing, editing.

Bobbi Pritt – writing, editing, provided pathology images.

Daniel DeSimone – writing, editing.

## Funding

None.

## Ethical approval

Not required.

## Consent

Obtained.

## Declaration of Competing Interest

The authors have no conflicts of interest to disclose
